# Effect of anxiolytic and hypnotic drug prescriptions on mortality hazards: retrospective cohort study

**DOI:** 10.1136/bmj.g1996

**Published:** 2014-03-19

**Authors:** Scott Weich, Hannah Louise Pearce, Peter Croft, Swaran Singh, Ilana Crome, James Bashford, Martin Frisher

**Affiliations:** 1 Division of Mental Health and Wellbeing, Warwick Medical School, University of Warwick, Coventry, West Midlands CV4 7AL, UK; 2 School of Pharmacy, Keele University, Keele, Staffordshire, UK; 3 Institute of Primary Care and Health Sciences, Keele University, Keele, Staffordshire, UK; 4 Academic Psychiatry Unit, St George’s Hospital, South Staffordshire and Shropshire Healthcare NHS Foundation Trust, Stafford, Staffordshire, UK

## Abstract

**Objective** To test the hypothesis that people taking anxiolytic and hypnotic drugs are at increased risk of premature mortality, using primary care prescription records and after adjusting for a wide range of potential confounders.

**Design** Retrospective cohort study.

**Setting** 273 UK primary care practices contributing data to the General Practice Research Database.

**Participants** 34 727 patients aged 16 years and older first prescribed anxiolytic or hypnotic drugs, or both, between 1998 and 2001, and 69 418 patients with no prescriptions for such drugs (controls) matched by age, sex, and practice. Patients were followed-up for a mean of 7.6 years (range 0.1-13.4 years).

**Main outcome** All cause mortality ascertained from practice records.

**Results** Physical and psychiatric comorbidities and prescribing of non-study drugs were significantly more prevalent among those prescribed study drugs than among controls. The age adjusted hazard ratio for mortality during the whole follow-up period for use of any study drug in the first year after recruitment was 3.46 (95% confidence interval 3.34 to 3.59) and 3.32 (3.19 to 3.45) after adjusting for other potential confounders. Dose-response associations were found for all three classes of study drugs (benzodiazepines, Z drugs (zaleplon, zolpidem, and zopiclone), and other drugs). After excluding deaths in the first year, there were approximately four excess deaths linked to drug use per 100 people followed for an average of 7.6 years after their first prescription.

**Conclusions** In this large cohort of patients attending UK primary care, anxiolytic and hypnotic drugs were associated with significantly increased risk of mortality over a seven year period, after adjusting for a range of potential confounders. As with all observational findings, however, these results are prone to bias arising from unmeasured and residual confounding.

## Introduction

Prescribing of hypnotic and anxiolytic drugs is common^1^ and increasing in places.^2^ In 2011-12 more than 16 million prescriptions for these drugs^3^ were written in general practice in England at a cost of over £60m ($100m; €73m) per annum. Benzodiazepines currently account for 62% and Z drugs (zaleplon, zolpidem, and zopiclone) 32% of total prescriptions for hypnotics and anxiolytics in primary care in England.^2^ ^4^

Evidence of adverse effects^5^ including increased risk of dementia^6^ ^7^ ^8^ and other psychomotor impairments (daytime fatigue, ataxia, falls, and road traffic incidents),^9^ ^10^ ^11^ ^12^ ^13^ ^14^ cancer,^15^ ^16^ ^17^ pneumonia, and other infections^18^ has increased concerns of an association with premature mortality. Until recently evidence for this was based on a small number of studies, which varied in setting, sample (especially age distribution), length of follow-up, source of drug usage data,^19^ ^20^ ^21^ type of drug, and the extent of control for confounding (especially from physical and psychiatric comorbidity, co-prescribing, socioeconomic status, smoking, and drug and alcohol misuse).^7^ ^22^ ^23^ Although two studies in older populations did not report a statistically significant association between benzodiazepine use and mortality after adjusting for confounders,^24^ ^25^ four others (in younger samples) found evidence of significantly increased mortality.^19^ ^20^ ^22^ ^23^ ^26^ A study in people with schizophrenia reported associations with suicide and with all cause mortality.^27^ Adjusted hazard ratios have varied substantially, ranging from 1.14^21^ to 4.56.^16^ ^23^ ^27^ A recent study^23^ found that the mortality risk extended to those with low levels of use, was greater in younger people, and that heavy use of hypnotics increased the risk of developing cancer.^23^ Questions remain about effect size, interactions with age,^20^ ^22^ ^24^ ^25^ and confounding (particularly by anxiety and other psychiatric disorders).

We tested the hypothesis that people taking anxiolytic or hypnotic drugs, or both, are at significantly increased risk of death compared with non-users and to estimate the size of this association after adjusting for a wide range of potential confounders using prescribing data from UK primary care.

## Methods

We undertook a retrospective, matched cohort study using the General Practice Research Database (GPRD). GPRD (incorporated into the Clinical Practice Research Datalink in 2012) was created in 1987 and is the largest anonymised, longitudinal primary care database in the world, with around 70 million patient years of high quality validated data from 630 practices. In 2011 over 11 million patient records were in the GPRD (five million active), equivalent to 8.3% of the UK population.^28^ The database contains records from clinical consultations with general practitioners, prescriptions, secondary care referrals, and hospital admissions.

This project was awarded a licence as part of a scheme operated by the UK Medical Research Council and Medicines and Healthcare products Regulatory Agency to provide data access on up to 100 000 patients. Data were based on records from 273 primary care practices in England, Scotland, Wales, and Northern Ireland.

### Participants

Eligible participants were patients aged 16 years and older, permanently registered with a practice contributing data to the GPRD, and with at least 12 months of up to standard records (as per GPRD data quality standards). We identified patients who had received study drugs by incident (first ever) prescription of an anxiolytic or hypnotic drug (see chapters 4.1.1 and 4.1.2 of the *British National Formulary*^29^), excluding barbiturates, during the recruitment window from January 1998 to December 2001. We only included patients who received at least two prescriptions for a given study drug during the recruitment period. This was done to minimise misclassification of use among people who received but did not fill the prescription or take the drug.^30^ ^31^ We reasoned that a second (that is, repeat) prescription indicated that the first had been filled and taken. Examination of a subsample of GPRD records (not reported here) found that 40.3% of people in receipt of a lifetime prescription for an anxiolytic or hypnotic drug only ever received a single prescription for that drug. No prescriptions for any study drugs were recorded for participants (whether users or non-users) for the duration of their practice record before recruitment into the study. Mean duration of registration with study practices before recruitment was 15.6 years (SD 14.0).

To improve efficiency and reduce the number of required patients who were prescribed the study drugs, we matched each patient prescribed any study drugs to two controls from among those with no prescription for any study drugs, on age (three years either way), sex, and practice. The 2:1 recruitment strategy was also determined by the 100 000 limit on the total sample size under the terms of our data license. Matching occurred (and follow-up started) at the time of the first prescription for a study drug. The period during which the study outcome (death) was ascertained therefore began at exactly the same time for both patients prescribed the study drugs and (matched) controls. Both groups of patients were followed until the earliest of death, censorship (no longer registered with practice), or truncation (end of the observation period on 31 October 2011). Study outcome was all cause mortality as recorded in the practice record. The observation period for the ascertainment of covariates was the entire interval for which data are available for a patient between the time their record starts (before recruitment) and either death, censorship, or truncation. To reduce the likelihood of bias arising from the prescription of study drugs to those who were terminally ill and nearing the end of life, we restricted the study sample further in our final model to patients who survived for longer than 12 months after recruitment.

### Ascertainment of study drug use

We ascertained the receipt of hypnotic and anxiolytic drugs from electronic prescribing records. Use was initially quantified in terms of defined daily doses from study entry point to the end of each patient’s observation period. The defined daily dose is the assumed average maintenance dose per day for a drug used for its main indication in adults (considered to be someone of 70 kg body weight).^32^ The defined daily dose, a measure of equivalence that permits pooling of usage data across different drugs used for the same indications and values, are available from the WHO Collaborating Centre for Drug Statistics Methodology (www.whocc.no/atc_ddd_index). The defined daily doses in the exposed group were recoded as a categorical variable: 1-30, 31-60, 61-90, and ≥91, corresponding to prescriptions of one, two, three, or more than three months’ duration. We classified study drugs as benzodiazepines, Z drugs (zaleplon, zolpidem, and zopiclone), and other. Patients who were prescribed study drugs were further dichotomised according to whether or not the study drug continued to be prescribed after the first 12 months of observation.

### Covariates and potential confounders

Statistical adjustment was undertaken for potential confounders. Controlling for confounding by indication (that is, possible reasons for being prescribed a study drug) is especially important. Potential confounders included sex, age at study entry, sleep disorders, anxiety disorders, other psychiatric disorders, medical morbidity, and prescriptions for non-study drugs. Smoking and alcohol use were recorded within the dataset as current, former, or never. As a means of controlling partially for differences in socioeconomic status, we matched patients who were or were not prescribed study drugs by practice.

Medical morbidity was ascertained using Read codes for arthritis and musculoskeletal problems, asthma, cancer, chronic obstructive pulmonary disease, diabetes, gastrointestinal disorders, epilepsy, hypertension, ischaemic heart disease, stroke, and sleep disorders (www.connectingforhealth.nhs.uk/systemsandservices/data/uktc/readcodes). We subdivided psychiatric codes into anxiety disorders (the main indication for anxiolytic prescribing) and all other psychiatric disorders.

### Data analysis

Using Cox proportional hazards models, we estimated the hazard ratios for death after recruitment into the study cohort (defined as the first prescription of a study drug). Exploratory analyses showed that the hazard function (for the association between study drugs and mortality) varied with age (results available from authors); we therefore stratified regression analyses by this variable.

In the first model we included prescriptions for all study drugs during the observation period (following recruitment) and included all deaths, regardless of timing. In the second model (to minimise confounding of use by survival) we restricted the exposed patient sample to those who were prescribed study drugs only in the first year after recruitment. All deaths were included, regardless of timing. In the third and final model, we further restricted both patient groups to those who survived for more than 12 months (and therefore after study drug prescription had ceased in the group using the study drugs).

We assessed the extent of co-prescribing of study drugs. Since 75.9% (n=26 347) of patients who used study drugs had received at least one prescription for a benzodiazepine and 31.5% (n=10 877) had received more than one class of study drug, we opted to pool estimates of association with mortality across groups in our primary analyses. We undertook subgroup analyses in which the group prescribed the study drugs was restricted to those who received benzodiazepines only, Z drugs only, or other study drugs only, in the first year after recruitment. MF and HP undertook all analyses by using SPSS version 19.0.

## Results

Data were obtained on 34 804 patients who were prescribed the study drugs and 69 585 patients (matched) who were not (controls). Seventy seven patients who were prescribed the study drugs were excluded (with 154 matched controls) owing to insufficient data to allow defined daily doses to be calculated. We also excluded 13 “unexposed” patients who had been prescribed melatonin during the observation period. The final sample for study models comprised 104 145 patients, of whom 34 727 were prescribed the study drugs and 69 418 were controls. Censorship (excluding death) was observed for 26.7% (n=9314) of the patients who were prescribed the study drugs and 31.2% (n=21 644) of controls (table 1).

**Table 1 tbl1:** Study drugs by drug class, showing defined daily doses (DDDs) and proportion of patients (n=34 727) who received a prescription for each of these during the observation period

Drug class and type	DDDs (mg)	No (% using drug)
Benzodiazepines:		
Alprazolam	1	19 ( 0.05)
Chlordiazepoxide	30	1788 (5.15)
Clobazam	20	164 (0.47)
Clonazepam	8	802 (2.31)
Diazepam	10	16 638 (47.91)
Flunitrazepam	1	3 (0.01)
Flurazepam hydrochloride	30	17 (0.05)
Loprazolam mesilate	1	161 (0.46)
Lorazepam	2.5	1472 (4.24)
Lormetazepam	1	665 (1.91)
Midazolam (including all salts)	15	523 (1.51)
Nitrazepam	5	1557 (4.48)
Oxazepam	50	597 (1.72)
Temazepam	20	12 208 (35.15)
Triazolam	0.25	0 (0)
Any benzodiazepine	—	26 347 (75.87)
Z drugs:		
Zaleplon	10	481 (1.39)
Zolpidem tartrate	10	2737 (7.88)
Zopiclone	7.5	11 764 (33.88)
Any Z drug	—	13 443 (38.71)
Other drugs:		
Alimemazine tartrate	30	331 (0.95)
Buspirone hydrochloride	30	916 (2.64)
Chloral hydrate	1000	72 (0.21)
Clomethiazole	1500	400 (1.15)
Clomethiazole edisilate	1500	82 (0.24)
Cloral betaine	1000	472 (1.36)
Dichloralphenazone	1300	0 (0)
Diphenhydramine hydrochloride	200	0 (0)
Hydroxyzine hydrochloride	75	2475 (7.13)
Melatonin	2	66 (0.19)
Meprobamate	1200	12 (0.03)
Methyprylone	200	0 (0)
Promethazine hydrochloride	25	1184 (3.41)
Sodium oxybate	7500	0 (0)
Triclofos sodium	1000	6 (0.02)
Any other drug	—	7445 (21.44)

Benzodiazepines (63.7% (n=22 116) of patients prescribed the study drugs) were more common as the index drug class than Z drugs (23.0%) (n=7971) or other study drugs (13.4%) (n=4640). Co-prescribing was common (table 2). In total, 76.3% (n=26 436) of patients using study drugs received a prescription for a benzodiazepine, 38.8% (n=13 444) a prescription for a Z drug, and 33.5% (n=7444) a prescription for one or more of the other study drugs. The most commonly prescribed study drugs were diazepam (47.9% of those prescribed the study drugs, n=16 638), temazepam (35.1%, n=12 208), and zopiclone (34.1%, n=11 764). Among the group prescribed the study drugs, 24.2% (n=8404) were only prescribed diazepam, 14.8% (n=5140) only temazepam, and 12.2% (n=4237) only zopiclone.

**Table 2 tbl2:** Patterns of prescribing and co-prescribing of study drugs at any time during observation period

Prescribed drug combinations	No (%) using drug
No co-prescribing:	
Benzodiazepines only	16 242 (46.8)
Z drugs only	4736 (13.5)
Other drugs only	2872 (8.3)
Co-prescribing from two classes:	
Benzodiazepines and Z drugs	6305 (18.2)
Benzodiazepines and other drugs	2169 (6.3)
Z drugs and other drugs	683 (2.0)
Co-prescribing of all drugs from all three classes:	
Benzodiazepines, Z drugs, and other drugs	1720 (5.0)
Total	34 727 (100)

Table 3 shows the characteristics of the study sample. Patients who were prescribed study drugs were more likely than controls to be current smokers and to have higher rates of all forms of physical morbidity, most notably cancer and respiratory disorders. The group prescribed study drugs also had higher rates of sleep (28.1% (n=9741) *v* 5.8% (n=4009)), anxiety (44.1% (n=15 299) *v* 11.3% (n=7849), and other psychiatric disorders (56.9% (n=19 770) *v* 21.7% (n=15 026)) than controls, and received more prescriptions for non-study drugs.

**Table 3 tbl3:** Characteristics of participants by use of study drugs. Values are percentages (numbers) unless stated otherwise

Characteristics	Study drug use		P value
	Users (n=34 727)	Non-users (n=69 418)	
Women	59.0 (20 492)	59.0 (40 964)	NS
Mean (SD) age at study entry (years)	55.5 (19.3)	55.4 (19.3)	NS
Mean (SD) duration of follow-up (months)	87.6 (51.1)	93.5 (48.5)	<0.001
Deaths during follow-up	26.5 (9190)	16.8 (11 678)	<0.001
Mean (SD) age at death (years)	76.2 (14.1)	82.1 (10.9)	<0.001
Smoking status:			
Not recorded	6.6 (2277)	10.2 (7105)	<0.001
Never smoker	45.8 (15 888)	53.6 (37 202)	
Current smoker	25.3 (8784)	15.0 (10 388)	
Former smoker	22.4 (7778)	21.2 (14 723)	
Alcohol status:			
Not recorded	14.2 (4915)	18.6 (12 897)	<0.001
Never drinker	18.3 (6342)	15.3 (10 617)	
Current drinker	64.1 (22 262)	64.5 (44 760)	
Former drinker	3.5 (1208)	3.4 (2352)	
Prescriptions:			
Median No (range) for study drug DDDs	70 (1-33 521)	0	—
Median No (range) of non-study drug prescriptions	227 (0-12 998)	83 (0-6435)	<0.001
Medical morbidity:			
Arthritis	25.5 (8853)	20.3 (14 110)	<0.001
Musculoskeletal problems	29.8 (28 621)	17.6 (6106)	<0.001
Asthma	17.3 (6003)	11.2 (7785)	<0.001
Cancer	23.7 (8233)	17.9 (12 422)	<0.001
Chronic obstructive pulmonary disease	15.3 (5298)	9.9 (6872)	<0.001
Gastrointestinal disorder	3.8 (1315)	2.2 (1526)	<0.001
Ischaemic heart disease	23.1 (8033)	18.1 (6274)	<0.001
Diabetes	13.8 (4778)	11.7 (8135)	<0.001
Epilepsy	5.1 (1757)	2.0 (1418)	<0.001
Hypertension	29.2 (10 141)	27.3 (18 964)	<0.001
Stroke	10.4 (3622)	7.3 (5061)	<0.001
Sleep disorder	28.1 (9741)	5.8 (4009)	<0.001
Anxiety disorder	44.1 (15 299)	11.3 (7849)	<0.001
Other psychiatric diagnoses	56.9 (19 770)	21.7 (15 026)	<0.001

NS=not significant; DDDs=defined daily doses.

We found statistically significant associations with mortality at all levels of study drug use. Our initial model classified use irrespective of when this occurred, and included all deaths regardless of when these occurred during the observation period (table 4). The hazard ratio for mortality in the group with the highest use of study drugs was lower than that in all three groups with fewer defined daily doses, suggesting that use was confounded by survival. We also noted reverse confounding on adjusting for study covariates.

**Table 4 tbl4:** Hazard ratios (95% confidence intervals) for age adjusted associations between defined daily doses (DDDs) of study drug (versus no study drugs) before and after adjusting for other potential confounders

Variables	No of patients	Age adjusted hazard ratio (95% CI)	P value	Fully adjusted* hazard ratio (95% CI)	P value
DDDs (all study drugs):					
0	69 418	1.00	—	1.00	—
1-30	10 275	1.89 (1.80 to 1.98)	<0.001	2.07 (1.97 to 2.17)	<0.001
31-60	6042	2.38 (2.25 to 2.51)	<0.001	2.58 (2.44 to 2.72)	<0.001
61-90	3207	2.39 (2.23 to 2.56)	<0.001	2.46 (2.29 to 2.63)	<0.001
≥91	15 203	1.77 (1.72 to 1.84)	<0.001	1.91 (1.84 to 1.98)	<0.001
Any DDDs	34 727	1.93 (1.87 to 1.98)	<0.001	2.08 (2.02 to 2.15)	<0.001

*Adjusted for age, sex, physical health problems (arthritis, asthma, cancer, ischaemic heart disease, stroke, chronic obstructive pulmonary disease, diabetes, epilepsy, gastrointestinal disorders, hypertension, musculoskeletal disorders, anxiety disorders, sleep disorders, other (non-anxiety), psychiatric disorders, and prescriptions for non-study drugs.

In the second model (table 5), the exposed group was restricted to those who received no prescriptions for the study drugs after their first year of observation. The age adjusted hazard ratio for mortality for any use of study drug was 3.46 (95% confidence interval 3.34 to 3.59), decreasing slightly to 3.32 (3.19 to 3.45) after adjusting for potential confounders. A clear dose-response association was found, with an adjusted hazard ratio for mortality of 4.51 (4.22 to 4.82) among those who received more than 90 defined daily doses of any study drug in the first year of follow-up. Associations with mortality, and dose-response effects, were found for each of the three separate classes of study drug. Hazard ratios were largest for benzodiazepines and smallest for other study drugs (table 5).

**Table 5 tbl5:** Hazard ratios (95% confidence interval) for age adjusted associations between defined daily doses (DDDs) of study drug (versus no study drugs) and mortality before and after adjusting for other potential confounders*, for exposure restricted to receipt of study drugs in first year after recruitment only

DDDs	No of patients	Age adjusted hazard ratio (95% CI)	P value	Fully adjusted* hazard ratio (95% CI)	P value
All study drugs:					
0	69 418	1.00	—	1.00	—
1-30	6648	2.47 (2.34 to 2.60)	<0.001	2.55 (2.42 to 2.69)	<0.001
31-60	2859	3.87 (3.66 to 4.15)	<0.001	3.78 (3.54 to 4.04)	<0.001
61-90	1185	5.04 (4.62 to 5.50)	<0.001	4.19 (3.84 to 4.58)	<0.001
≥91	1971	5.59 (5.21 to 5.92)	<0.001	4.51 (4.22 to 4.82)	<0.001
Any DDDs	12 663	3.46 (3.34 to 3.59)	<0.001	3.32 (3.19 to 3.45)	<0.001
Benzodiazepines only:					
1-30	4625	2.71 (2.54 to 2.88)	<0.001	2.78 (2.62 to 2.96)	<0.001
31-60	1414	5.17 (4.77 to 5.59)	<0.001	4.62 (4.26 to 5.01)	<0.001
61-90	549	6.62 (5.91 to 7.42)	<0.001	5.42 (4.84 to 6.08)	<0.001
≥91	881	6.75 (6.18 to 7.34)	<0.001	5.10 (4.67 to 5.58)	<0.001
Any DDDs	7469	3.89 (3.73 to 4.06)	<0.001	3.68 (3.52 to 3.85)	<0.001
Z drugs only:					
1-30	858	2.39 (2.07 to 2.77)	<0.001	2.36 (2.05 to 2.74)	<0.001
31-60	807	3.53 (3.08 to 4.04)	<0.001	3.41 (2.98 to 3.91)	<0.001
61-90	278	4.58 (3.75 to 5.59)	<0.001	3.66 (2.99 to 4.48)	<0.001
≥91	492	4.83 (4.22 to 5.54)	<0.001	3.90 (3.39 to 4.47)	<0.001
Any DDDs	2435	3.50 (3.24 to 3.77)	<0.001	3.19 (2.95 to 3.45)	<0.001
Other study drugs only:					
1-30	976	1.93 (1.71 to 2.19)	<0.001	1.86 (1.64 to 2.10)	<0.001
31-60	378	2.02 (1.67 to 2.43)	<0.001	2.09 (1.73 to 2.51)	<0.001
61-90	154	2.92 (2.25 to 3.82)	<0.001	2.46 (1.87 to 3.22)	<0.001
≥91	175	3.34 (2.68 to 4.16)	<0.001	2.65 (2.13 to 3.30)	<0.001
Any DDDs	1683	2.18 (2.00 to 2.38)	<0.001	2.06 (1.88 to 2.25)	<0.001

*Age, sex, physical health problems (arthritis, asthma, cancer, ischaemic heart disease, stroke, chronic obstructive pulmonary disease, diabetes, epilepsy, gastrointestinal disorders, hypertension, musculoskeletal disorders, anxiety disorders, sleep disorders, other (non-anxiety), psychiatric disorders, and prescriptions for non-study drugs.

We further excluded patients in both groups with less than one year of follow-up (model 3, table 6). Those who survived the first year but did not receive prescriptions for the study drugs beyond the first year of observation are the subgroup analysed in table 6. They had lower rates of physical and mental health problems on all 14 indicators of comorbidity than those who were prescribed study drugs beyond the first year, including cancer (22.9% (n=5050) *v* 18.6% (n=1599)), chronic obstructive pulmonary disease (15.7% (n=3449) *v* 13.5% (n=1158)), and ischaemic heart disease (22.5% (n=4955) *v* 19.6% (n=1683)). Those only prescribed study drugs in year 1 also differed slightly from those who took study drugs beyond the first year on mean age at study entry (52.6 years (SD 18.9) *v* 54.3 years (SD 18.6), P<0.001) and age at death (77.2 years (SD 13.8) *v* 76.8 years (SD 14.3), P=0.38). Patients who were only prescribed study drugs in year 1 were less likely to die than those who continued to take drugs (18.8% (n=1610) *v* 22.0% (n=4852)) but more likely to be censored for other reasons (33.5% (n=3028) *v* 22.3% (n=4906)).

**Table 6 tbl6:** Hazard ratios (95% confidence intervals) for age adjusted associations between defined daily doses (DDDs) of study drug and mortality before and after adjusting for other potential confounders, for exposure restricted to receipt of study drugs in first year after recruitment only and for patients with at least 12 months of follow-up

DDDs	No of patients	Age adjusted hazard ratio (95% CI)	P value	Fully adjusted* hazard ratio (95% CI)	P value
All study drugs:					
0	63 717	1.00	—	1.00	—
1-30	5142	1.46 (1.35 to 1.57)	<0.001	1.45 (1.35 to 1.56)	<0.001
31-60	1873	2.02 (1.82 to 2.23)	<0.001	1.94 (1.76 to 2.16)	<0.001
61-90	659	2.27 (1.94 to 2.66)	<0.001	1.87 (1.59 to 2.19)	<0.001
≥91	910	3.14 (2.80 to 3.52)	<0.001	2.63 (2.34 to 2.95)	<0.001
Any DDDs	8584	1.83 (1.73 to 1.92)	<0.001	1.75 (1.65 to 1.85)	<0.001
Benzodiazepines only:					
1-30	3561	1.46 (1.33 to 1.59)	<0.001	1.47 (1.34 to 1.61)	<0.001
31-60	791	2.31 (2.01 to 2.65)	<0.001	2.07 (1.80 to 2.38)	<0.001
61-90	251	2.63 (2.09 to 3.32)	<0.001	2.31 (1.84 to 2.92)	<0.001
≥91	361	4.14 (3.53 to 4.87)	<0.001	3.32 (2.83 to 3.89)	<0.001
Any DDDs	4964	1.88 (1.76 to 2.02)	<0.001	1.81 (1.68 to 1.94)	<0.001
Z drugs only:					
1-30	655	1.39 (1.12 to 1.71)	<0.001	1.34 (1.08 to 1.66)	<0.001
31-60	603	2.05 (1.67 to 2.51)	<0.001	2.05 (1.67 to 2.51)	<0.001
61-90	186	2.43 (1.76 to 3.36)	<0.001	1.81 (1.31 to 2.51)	<0.001
≥91	271	2.7 (2.15 to 3.39)	<0.001	2.25 (1.79 to 2.84)	<0.001
Any DDDs	1715	1.94 (1.72 to 2.17)	<0.001	1.78 (1.58 to 2.01)	<0.001
Other study drugs only:					
1-30	785	1.5 (1.28 to 1.75)	<0.001	1.45 (1.24 to 1.69)	<0.001
31-60	310	1.58 (1.26 to 2.01)	<0.001	1.65 (1.31 to 2.09)	<0.001
61-90	113	1.93 (1.32 to 2.84)	<0.001	1.75 (1.19 to 2.57)	<0.001
≥91	109	2.36 (1.73 to 3.25)	<0.001	1.9 (1.39 to 2.61)	<0.001
Any DDDs	1317	1.63 (1.45 to 1.82)	<0.001	1.57 (1.40 to 1.76)	<0.001

*Age, sex, physical health problems (arthritis, asthma, cancer, ischaemic heart disease, stroke, chronic obstructive pulmonary disease, diabetes, epilepsy, gastrointestinal disorders, hypertension, musculoskeletal disorders, anxiety disorders, sleep disorders), other (non-anxiety) psychiatric disorders, and prescriptions for non-study drugs.

Patterns of association remained in this third model, although effect sizes were reduced (adjusted hazard ratio for >90 defined daily doses was reduced to 2.63 (95% confidence interval 2.34 to 2.95), table 6). The same patterns of association were found across all three classes of study drugs, with hazard ratios for benzodiazepines being the largest. The adjusted hazard ratio for use of any drug in model 2 (table 5), which included early deaths during drug use, was 3.32 (95% confidence interval 3.19 to 3.45), compared with an adjusted hazard ratio for any drug use in model 3, limited to deaths after prescriptions for the study drugs had finished, of 1.75 (1.65 to 1.85, table 6).

## Discussion

We found evidence of an association between prescription of anxiolytic and hypnotic drugs and mortality over an average follow-up period of 7.6 years among more than 100 000 age and general practice matched adults. In patients who were prescribed these drugs, there was an estimated overall statistically significant doubling of the hazard of death (hazard ratio 2.08), after adjusting for a wide range of potential confounders, including physical and psychiatric comorbidities, sleep disorders, and other drugs. This association remained significant and followed a dose-response pattern after restricting analyses to those with at least 12 months of follow-up and to those who were only prescribed the study drugs in the first year after recruitment (hazard ratio 1.75). Crude cumulative mortality in those given drugs was 26.46 per 100 people over the full follow-up period compared with 16.82 per 100 controls. After excluding deaths in the first year, there were approximately four excess deaths linked to drug use per 100 people followed for an average of 7.6 years after their first prescription.

While overall effect sizes were broadly in keeping with most previous findings, our estimates of association were lower than that reported by one study,^23^ which reported an adjusted hazard ratio of 4.56 over 2.5 years. This may reflect differences in the length of follow-up, as both studies reported declining associations with mortality over time.

### Strengths and limitations of this study

Use of data from the UK General Practice Research Database was an obvious strength, given the size and representativeness of the sample, and the quality, completeness, and duration of the follow-up data. Data on drug use were based on documented prescriptions rather than self reported receipt or use of drugs. We had detailed information on a wide range of potential confounders, going back several years. In particular, we were able to control for a large number of physical and psychiatric morbidities as well as prescriptions of other drugs. This is especially important given the possibility of confounding by indication (that is, study drugs may be given more often to those who are seriously ill and who may not be able to sleep because of pain or other consequences of long term or life threatening illnesses). In contrast with a recent report,^23^ we were able to adjust for anxiety disorders as well as all other psychiatric disorders. We were also able to identify and control for recorded instances of sleep problems (including those secondary to physical and psychiatric disorders).

Our recruitment strategy and ascertainment of drug use were further strengths. We minimised misclassification by excluding people who had received only one prescription, since some people never fill prescriptions or take the drugs. Using defined daily doses to quantify cumulative use of study drugs allowed us to combine the effects of different drugs in a way that is not possible by counting prescriptions or pills.^23^ In further contrast with previous research,^23^ we chose to classify study drugs by class rather than by indication for the purposes of recruitment; for example, we included all benzodiazepines, not just those recorded as having been prescribed for insomnia. We would argue that this resulted in a more accurate estimation of use of these drugs, as well as ensuring that our results are generalisable to all of those who receive anxiolytic and hypnotic drugs in primary care. Our models were adjusted for all main indications for these drugs. Although pooling of study drugs may have overlooked variation in associations with mortality across classes, subgroup analyses indicated statistically significant associations (and dose-response effects) between mortality and all three classes of study drug. The largest hazard ratios were found for benzodiazepines.

Despite using prescribing records, we may have underestimated use of study drugs. Patients with more serious psychiatric disorders may be cared for by secondary care services rather than solely in primary care. Although in most cases responsibility for longer term (repeat) prescribing is usually delegated to general practice, it is possible that prescribing for these patients may be under-recorded in the General Practice Research Database. Likewise, we had no information on the use of study drugs that were obtained illicitly, although this was likely to have been modest compared with use of prescribed drugs. It is highly unlikely that study drugs were used before recruitment among the patients eventually prescribed the drugs or controls, given that the patients were registered with the study practices for 15 years on average. This was not so in a previous study,^23^ in which around one fifth of the exposed group had received a prescription for a study drug before recruitment.

The length of follow-up was also a strength, particularly for the generalisability of the findings. However, higher effect estimates obtained when we restricted our sample of exposed patients to those with no further prescriptions for the study drugs after the first 12 months suggests that results from our initial model, which included patients who continued to receive prescriptions for the study drugs throughout the observation period, may have been biased towards the null by the confounding of use and survival. Results of models in which use was restricted to the first year after recruitment and deaths restricted to those occurring after that first year suggest that much of the excess mortality risk arises early in the period of drug use but remains statistically significant even after discontinuing study drugs. We were not, however, able to explore temporal risk trajectories in detail. It was possible that patients who discontinued drugs within the first year did so because they were particularly unwell (and more morbid than those who continued to take these drugs). However, our findings show that the opposite was true, which strengthens the validity of our estimates of excess mortality in this group compared with the control group. Although those for whom prescriptions for study drugs stopped after the first year were more likely to be censored for reasons other than death, there is no reason to believe that this inflated the association between study drugs and mortality.

Non-randomised outcome studies are especially prone to confounding, including confounding by indication. One option for dealing with confounding by indication is using a comparator group more closely aligned with the exposed group—for example, patients who were starting other types of drugs. However, in the absence of previous evidence that comparator drugs were free of other indication effects, this would again have not ruled out this bias, even though it may have accounted for bias related to the comparison with non-users. Instead, we chose to deal with this in four ways: by taking account of a large number of potential confounders, by comparing effects across different groups of study drugs, by conducting subgroup analyses that limited exposure to year 1 and excluded all deaths during that first year in patients who both used and did not use the study drugs (on the grounds that confounding by indication will have the largest effect in year 1), and by adjusting our estimates of association for comorbidities occurring across the entire follow-up period. Nevertheless, although we controlled for many potential factors that were associated with study drug use and mortality and eliminated confounding of use and survival, it is impossible to exclude confounding arising from unmeasured factors or measurement error.^33^ ^34^ ^35^ While effects on estimates of association can be substantial,^33^ such bias is greatest for unmeasured confounders and those that are uncorrelated with other confounders but correlated with the study exposure.^34^ Bias tends to be greatest in studies that control for relatively few measured confounders.^34^

Although bias due to confounding was likely to have occurred, the impact was offset by the large number of covariates included in our analyses. One important unmeasured confounder is socioeconomic status, since records in the General Practice Research Database do not include detailed information on occupation, education, housing tenure, income, or employment. However, this variable was partially controlled for by matching by practice. Residual confounding, arising from a mixture of misclassification and indication, was also likely to have occurred in the recording of clinical diagnoses, and through our inability to quantify the severity of illness. Again, this is likely to have been offset to an extent by controlling for a wide range of comorbidities. Adjustment for a large number of measured confounders failed to negate our finding of an association between drug use and mortality but has resulted in appropriately more conservative estimates of the size of this association.^23^

Cohort studies are also prone to immortal time bias, which arises if the period participants are considered at risk differs between comparator groups.^36^ Although mortality was counted from the time of the first prescription for all patients, the time until second prescription would be “immortal” for patients who used the study drugs and who had to survive to get a second prescription to be in the study. This would not have applied to controls. However, excluding deaths among those who did not survive to receive a second prescription would have underestimated mortality in patients who used the study drugs. Any bias in the mortality comparison would therefore have been towards the null. Furthermore, this would not have biased the subgroup analysis that excluded early deaths, since deaths were ascertained only after the first year of follow-up for patients who both did and did not use the study drugs.

We considered the possibility of collider bias, a form of selection bias that may occur when two variables are not associated but share a common antecedent or outcome. Adjusting for such a factor can result in a spurious association.^37^ It is possible, for example, that study drug use and mortality are both associated with (for example) physical illness but themselves are not related. Since adjusting for comorbidities reduced estimates of association between study drugs and mortality, we suggest that these variables were likely to have been acting as classic confounders rather than sources of selection bias.

We did not have access to data on cause of death and therefore were unable to explore associations between prescription of study drugs and specific forms of morbidity such as pneumonia. Neither did we explore interactions between individual forms of morbidity and vulnerability to specific drug classes. In the light of consistent evidence of associations with mortality, such investigations are needed and will be the subject of future studies.

### Conclusions

These findings are consistent with previous evidence of a statistically and clinically significant association between anxiolytic and hypnotic drugs and mortality. Using prescribing data from a large primary care database and after adjusting for a wide range of potential confounders, prescriptions for these drugs were associated with significantly increased risks of mortality over an average follow-up period of 7.6 years. This association followed a dose-response pattern for all three classes of study drug and extended beyond the time of use. However, as with all observational studies, these findings remain prone to many forms of bias. While we have largely excluded immortal time bias and selection bias, we are unable to exclude the possibility that the results were due to confounding by indication or to residual confounding by unmeasured or incompletely measured factors, such as socioeconomic status. This applies especially to deaths in the first year of observation. These results add to evidence of an association with mortality, but must be treated with caution.

**What is already known on this topic**

Anxiolytic and hypnotic drugs are addictive and associated with cognitive and psychomotor impairments, falls, and unintentional injuries
It has long been suspected that these drugs are associated with premature mortality
Studies of the association between anxiolytic and hypnotic drugs and mortality have reported widely varying effect sizes, reflecting variation in methods

**What this study adds**

In this large cohort of patients attending UK primary care, anxiolytic and hypnotic drugs were associated with a significantly increased risk of mortality over a seven year period
After excluding deaths in the first year, there were about four excess deaths linked to drug use per 100 people followed for an average of 7.6 years after their first prescription
The risk followed a dose-response pattern and was found for all three classes of study drugs and did not seem to be entirely due to confounding by physical or psychiatric comorbidity or prescribing of other drugs
This study controlled for a wide range of potential confounders including the major sources of confounding by indication

## Notes

This study is based on data from the Full Feature General Practice Research Database obtained under licence from the UK Medicines and Healthcare Products Regulatory Agency (MHRA). However, the interpretation and conclusions contained in this study are those of the authors alone. Access to the General Practice Research Database was funded through the Medical Research Council’s licence agreement with MHRA. We thank Tarita Murray-Thomas at the MHRA for producing the dataset, and all contributing general practitioners and their patients. PC is a senior investigator for the National Institute for Health Research.

Contributors: SW, MF, SS, and IC had the original idea for this study. SW and MF were responsible for study hypotheses, design, data specification, analysis, and drafting of the manuscript. MF and HP undertook the analyses, and results were interpreted by all authors. PC advised on data analysis and interpretation of the study findings. MF had full access to all the data in the study and takes responsibility for the integrity of the data and the accuracy of the data analysis. All authors contributed to the drafting of the manuscript. SW is the guarantor.

Ethical approval: This project was approved by the Warwick Medical School Biomedical Research Ethics Committee (reference 192-03-2012).

Data sharing: No additional data available. The study data remain the property of the Clinical Practice Research Datalink (formerly General Practice Research Database) and was provided to the authors under license.

Transparency: SW (the manuscript’s guarantor) affirms that the manuscript is an honest, accurate, and transparent account of the study being reported; that no important aspects of the study have been omitted; and that any discrepancies from the study as planned (and, if relevant, registered) have been explained.

## Notes

Cite this as: *BMJ* 2014;348:g1996
